# Inhibition of Virus-Induced Cytokine Production from Airway Epithelial Cells by the Late Addition of Budesonide

**DOI:** 10.3390/medicina56030098

**Published:** 2020-02-27

**Authors:** Tetsuya Homma, Yosuke Fukuda, Yoshitaka Uchida, Tomoki Uno, Megumi Jinno, Yasunari Kishino, Mayumi Yamamoto, Hiroki Sato, Kaho Akimoto, Keisuke Kaneko, Akiko Fujiwara, Haruna Sato, Kuniaki Hirai, Yoshito Miyata, Hideki Inoue, Shin Ohta, Yoshio Watanabe, Sojiro Kusumoto, Koichi Ando, Shintaro Suzuki, Toshimitsu Yamaoka, Akihiko Tanaka, Tohru Ohmori, Hironori Sagara

**Affiliations:** Department of Medicine, Division of Allergology and Respiratory Medicine, Showa University School of Medicine, Tokyo 142-8555, Japan; terubow0423@gmail.com (Y.F.); for.u.goodlife@gmail.com (Y.U.); unouno0926@gmail.com (T.U.); jinno@med.showa-u.ac.jp (M.J.); ookiyookiy@med.showa-u.ac.jp (Y.K.); ymym0018@med.showa-u.ac.jp (M.Y.); sato_hiro@med.showa-u.ac.jp (H.S.); k_akimoto@med.showa-u.ac.jp (K.A.); lumiere.b.324@gmail.com (A.F.); rainbow@med.showa-u.ac.jp (H.S.); medi123@infoseek.jp (K.H.); fourdimensions28@gmail.com (Y.M.); hiyumi2001@gmail.com (H.I.); shinohta@med.showa-u.ac.jp (S.O.); fzr02034@nifty.ne.jp (Y.W.); k-sojiro@med.showa-u.ac.jp (S.K.); koichi-a@med.showa-u.ac.jp (K.A.); szshintr@med.showa-u.ac.jp (S.S.); toshimitsu.yamaoka@gmail.com (T.Y.); tanakaa@med.showa-u.ac.jp (A.T.); ohmorit@med.showa-u.ac.jp (T.O.); sagarah@med.showa-u.ac.jp (H.S.)

**Keywords:** budesonide, thymic stromal lymphopoietin (TSLP), rhinovirus, epithelial cells

## Abstract

*Background and objectives*: Viral infection is the main cause of asthma and COPD (chronic obstructive pulmonary disease) exacerbation and accumulate inflammatory cells to airway tissue. We have reported poly I:C, a mimic product of the virus and ligand of toll-like receptor 3 (TLR3), induced inflammatory chemokines from airway epithelial cells and found prior incubation with corticosteroids diminishes the effect of TLR3 activation. In clinical practice, mild asthma is recommended as-needed budesonide (BUD) when symptoms occur following a viral infection, etc. However, many questions still surround BUD’s usefulness if taken after a virus has already infected airway tissue. The aim of this study was to investigate the inhibitory effects of BUD on inflammatory cytokines induced by viral infection. *Materials and Methods*: Normal human bronchial epithelial (NHBE) cells were stimulated with poly I:C or infected with human rhinovirus-16 (HRV16) and BUD was added after the initial stimulation. Expression of both thymic stromal lymphopoietin (TSLP) and CCL26/eotaxin-3 was quantified by real-time RT-PCR and enzyme-linked immunosorbent assay (ELISA), respectively. Knockdown study was performed. *Results*: Pre-or post-incubation with BUD inhibited both poly I:C- and HRV16-induced mRNAs and proteins of both thymic stromal lymphopoietin (TSLP) and CCL26 with significance. Knockdown of the glucocorticoid receptor diminished these effects of BUD. Under the same conditions of BUD’s experiment, post-incubation with neither fluticasone propionate nor dexamethasone suppressed expression of both TSLP and CCL26, which induced by poly I:C. *Conclusion*: Post-addition of BUD inhibited the virus-induced TSLP and CCL26 from the airway epithelial cells. These results suggest that inhalation of BUD after viral infection has beneficial effects on asthma. Conclusion: Late addition of BUD may benefit among patient with viral infection and type 2 allergic airway disease such as asthma.

## 1. Introduction

Type 2 allergic inflammation, such as bronchial asthma and chronic rhinosinusitis (CRS), are major health concerns around the world [1,2,3]. The World Health Organization estimated that asthma relates to 1 in every 250 deaths around the world [4]. The approximate number of prevalence of adult asthma in developed countries is 10% and prevalence is lower in developing countries, but importantly increasing [5].

Epithelial cells are known to play a key role in innate immune responses against viral pathogens and to modulate the responses to inhaled allergens [1,6,7,8,9]. Jackson and colleagues recently reported the relationships between the virus and the pathogenesis of persistent Th2-related airway diseases, such as asthma [10]. Our recent publication described the inhibition of poly I:C signaling by a glucocorticosteroid in bronchial epithelium, resulting in reduced induction of inflammatory chemokines and cytokines, such as eosinophil-related CCL26/eotaxin-3 and neutrophil-related CXCL8/IL-8 [11]. Inhibition of viral-signaling by a glucocorticosteroid in the epithelium is especially critical in clinical practice [12]. Viral recognition receptors, such as toll-like receptor 3 (TLR3) expressed by epithelial cells, are activated to produce inflammatory cytokines and chemokines, including thymic stromal lymphopoietin (TSLP), CCL26, and CXCL8 when viral infection occurs [13,14]. Importantly these reactions are inhibited by a glucocorticosteroid [13,14].

Viral infection is one of the greatest causes of asthma attacks encountered in clinical practice, which is normally treated with a glucocorticosteroid. In mild asthma, as-needed use of budesonide (BUD)/formoterol (FOR) (Symbicort^®^) is recommended for the relief of symptoms triggered by viral infections. 

However, there is little evidence from basic research on the usefulness of a glucocorticosteroid when administered after a viral infection. In this study, we investigated the benefits of BUD in inhibiting the production of inflammatory cytokines induced by viral infection.

## 2. Methods

### 2.1. Reagents

Poly I:C (InvivoGen, San Diego, CA, USA), IL-13, IL-17A, budesonide (BUD), fluticasone propionate (FP), and dexamethasone (DEX) (R&D Systems, Minneapolis, MN, USA) were purchased from listed suppliers. Small interfering RNA (siRNA) against glucocorticoid receptor (GCR) and the no-effect control (Santa Cruz Biotechnology, Santa Cruz, CA, USA) and HiPerFect transfection reagent (Qiagen, Valencia, CA, USA) were purchased from listed suppliers as well.

Cell Culture, Transfection, Treatments, and Human Rhinovirus16 Infection.

Primary normal human bronchial epithelial (NHBE) cells (Lonza, Walkersville, MD, USA) from at least four donors were plated in 12-well culture plates. Hydrocortisone deprived medium was used for 24 h before any treatment to NHBE cells. NHBE cells were treated with BUD, FP, or DEX 1 to 3 h before cell harvest or 1 hour before stimulation with poly I:C (5 μg/mL), IL-13 (100 ng/mL), or IL-17A (100 ng/mL) for 6 to 24 h. NHBE cells were transfected with siRNA (10 nM) against nonspecific control or GCR at 50% confluency using HiPerFect transfection reagent as described previously [14]. HRV16 stocks were amplified and purified based on the published protocol [15]. NHBE cells grown in 12-well culture plates and were infected with HRV16 at a multiplicity of infection of 1 (MOI = 1). HRV16-infected NHBE cells were cultured for 24 h at 33 °C in the presence or absence of corticosteroids, BUD, FP, and DEX. Each experiment was performed more than four times.

### 2.2. Real-Time RT-PCR

cDNA was synthesized from isolated total RNA from the cells and real-time RT-PCR was performed as described previously [14,16]. β-actin was used as the housekeeping gene to normalize the levels of expression of mRNA. The sequences of primers and probe sets for detection of β-actin (PPH00073G-200), TSLP (PPH18939B-200), CCL26 (PPH01163F-200), CXCL8 (PPH00568A), and glucocorticoid-induced leucine zipper (GILZ) (PPH02879B-200) were purchased from QIAGEN.

### 2.3. ELISA

Commercially available ELISA kits (Minneapolis, MN, USA) were used to determine the expressed levels of TSLP, CCL26, and CXCL8 in the supernatants of cultured cells with a following the manufacturer’s instructions (R&D Systems).

### 2.4. Statistical Analysis

All data are presented as the mean ± SEM unless otherwise mentioned. Data were normally distributed, and differences between groups were analyzed using the paired Student’s *t*-test, with *p* < 0.05 considered to be statistically significant. All statistical analyses were performed using GraphPad Prism 5.0 software (GraphPad Software, La Jolla, CA, USA).

### 2.5. Ethics Statement

The approval number was 2401 and the approval date was 14 December 2017 by the ethic committee.

## 3. Results

**Figure 1:**
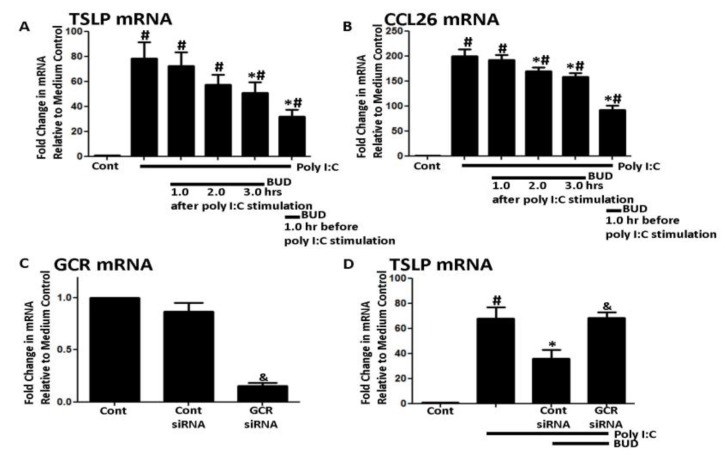
(**A**,**B**) Time course of suppression of poly I:C-induced thymic stromal lymphopoietin (TSLP) and CCL26 mRNA by budesonide. The poly I:C (5 ug/mL)-induced TSLP and CCL26 mRNA were suppressed when budesonide (10^−7^ M) was added 3 hours prior to harvest or pre-applied before poly I:C stimulation. (**C**,**D**) Glucocorticoid receptor (GCR) was knocked down by the siRNA method and the cells were stimulated with poly I:C (5 ug/mL). (**D**) Budesonide (10^−7^ M) was added 1 hour prior to poly I:C stimulation and the suppressive effect of budesonide was diminished. Data represent mean ± SEM of four independent experiments. # *p* < 0.01 by *t*-test when compared with medium-only control. * *p* < 0.01 by *t*-test when compared with poly I:C-treated cells and *p* < 0.01 by *t*-test when compared with control siRNA-transfected cells. & *p* < 0.01 by *t*-test when compared with control siRNA transfected cells.

Poly I:C concentration was set at 5 ug/mL as in past studies of ours [16,17]. NHBE cells expressed TSLP and CCL26 mRNA after six hours of poly I:C stimulation (Figure 1A,B). Both TSLP and CCL26 mRNA production were inhibited by BUD (10^−7^ M) applied one hour before poly I:C stimulation or 3 h before cell harvest (Figure 1A,B). Next, GCR or control siRNA was transfected to NHBE cells and the knockdown efficiency was more than 90% (Figure 1C). Then, the transfected cells were exposed to BUD followed by poly I:C. The suppressive effect of BUD was diminished by GCR knockdown (Figure 1C,D). 

**Figure 2:**
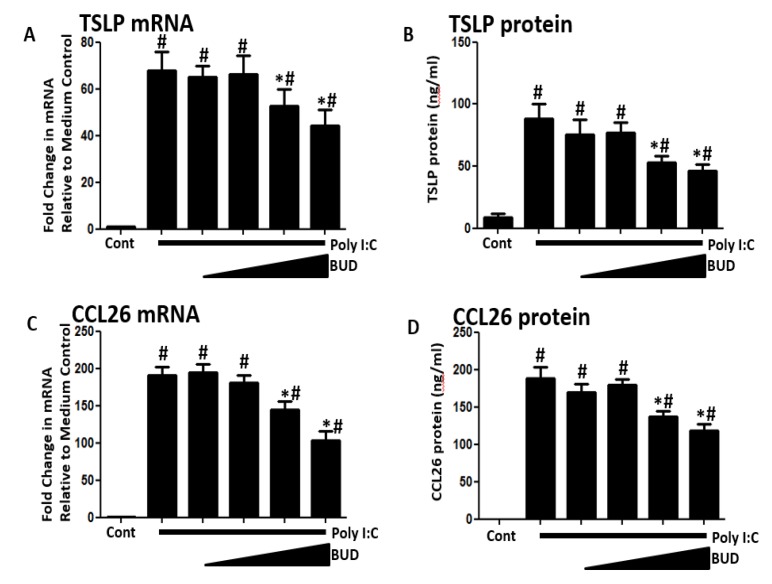
Poly I:C (5 ug/mL)-induced (**A**,**B**) TSLP and (**C**,**D**) CCL26 was suppressed by budesonide in a dose-dependent manner. The used budesonide concentration was 10^−6^, 10^−7^, 10^−8^, and 10^−9^ M. Data represent mean ± SEM of four independent experiments. # *p* < 0.01 by *t*-test when compared with medium-only control. * *p* < 0.01 by *t*-test when compared with poly I:C-treated cells.

BUD′s effects were tested at a range of concentrations (10^−6^ to 10^−9^ M), and then, 10^−7^ M was selected for subsequent experiments because levels of both TSLP and CCL26 mRNA and protein expression were significantly suppressed at all concentrations ≥10^−7^ M (Figure 2A–D). 

**Figure 3:**
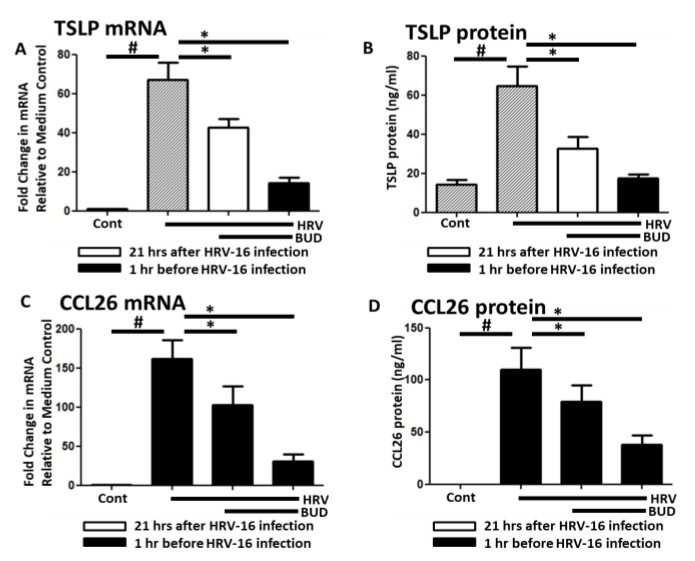
Both TSLP and CCL26 were suppressed by the late addition of budesonide. (**A**,**B**) HRV16 (MOI = 1)-induced both TSLP mRNA and protein which were suppressed by the prior or late addition of budesonide (10^−7^ M). (**C**,**D**) CCL26 mRNA and protein were also decreased in the same trend as TSLP mRNA and protein, respectively. Data represent mean ± SEM of four independent experiments. # *p* < 0.01 by *t*-test when compared with medium-only control. * *p* < 0.01 by *t*-test when compared with poly I:C-treated cells.

HRV-16 (MOI = 1) infection induced both TSLP and CCL26 mRNA and protein expression in NHBE cells. Similar to the poly I:C tests, these expressions were significantly inhibited by 10^−7^ M BUD applied 1 h before infection or 3 h before cell harvest (Figure 3).

**Figure 4:**
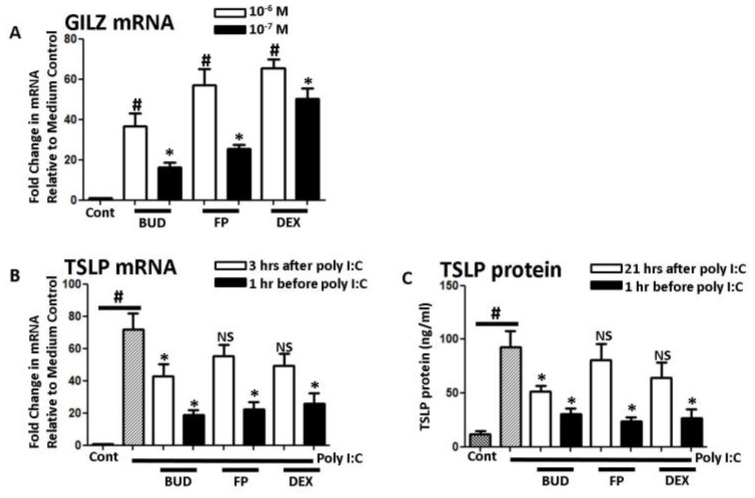
Poly I:C (5 ug/mL)-induced TSLP by other corticosteroids. (**A**) Stimulation with either budesonide (BUD) (10^−7^ or 10^−6^ M), fluticasone propionate (FP) (10^−7^ or 10^−6^ M), or dexamethasone (DEX) (10^−7^ or 10^−6^ M)-induced glucocorticoid-induced leucine zipper (GILZ) mRNA. (**B**,**C**) TSLP mRNA and protein were not inhibited when FP and DEX were added 3 h prior for harvest, while adding BUD, FP, and DEX before poly I:C stimulation inhibited the TSLP induction. Data represent mean ± SEM of four independent experiments. # *p* < 0.01 by *t*-test when compared with medium-only control. * *p* < 0.01 by *t*-test when compared with poly I:C-treated cells. NS: not significant when compared with poly I:C-treated cells.

Glucocorticoid-induced leucine zipper (GILZ) activity was induced by all glucocorticosteroid species (Figure 4A). Similar to BUD, FP and DEX inhibited TSLP mRNA and protein production when applied before poly I:C stimulation; however, FP and DEX did not inhibit TSLP mRNA and protein when applied three hours before cell and supernatant collection (Figure 4B,C).

**Figure 5:**
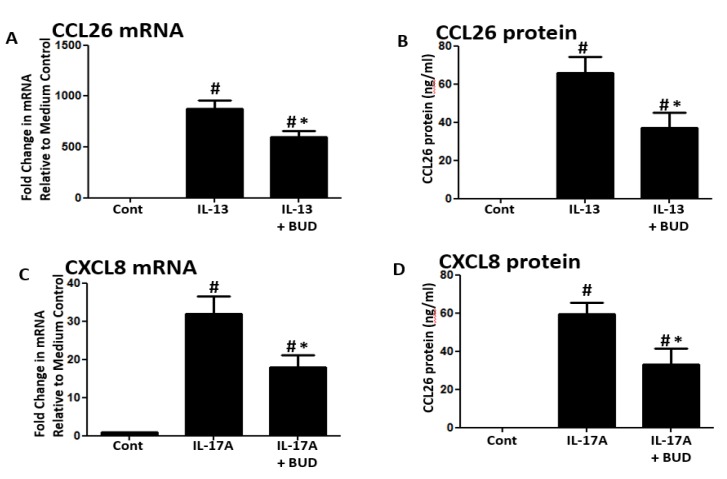
Inflammatory cytokine-induced genes by the late addition of budesonide. (**A**,**B**) IL-13 (100 ng/mL)-induced CCL26 mRNA and protein were suppressed by the addition of budesonide (10^−7^ M) 3 h before the harvest. (**C**,**D**) IL-17A (100 ng/mL)-induced CXCL8 mRNA and protein were suppressed by the addition of budesonide 3 hours before the harvest. Data represent mean ± SEM of four independent experiments. # *p* < 0.01 by *t*-test when compared with medium-only control. * *p* < 0.01 by *t*-test when compared with IL-13- or IL-17A -treated cells.

Next, we investigated BUD’s effect on two cytokines crucial to the pathogenesis of asthma and CRS. Stimulation with IL-13 induced the expression of CCL26 mRNA and protein in NHBE cells, but their expression was significantly suppressed when BUD was applied 3 h before cell and supernatant collection (Figure 5A,B). Similarly, IL-17A stimulation induced the production of CXCL8 mRNA and protein, but levels were much lower when BUD was applied 3 h before cell and supernatant collection (Figure 5C,D).

## 4. Discussion

Our results showed that BUD significantly inhibited poly I:C-induced TSLP expression whether added before or after poly I:C exposure, and similar effects were also observed in response to HRV16 infection. These effects were mediated by GCR, identical to other glucocorticosteroids. Skevaki et al. showed the inhibition of virus-induced chemokines and cytokines by the late addition of BUD and showed the suppression of expressed proteins, which supports our data [18]. Interestingly, FP and DEX failed to show the same inhibitory effects when applied after poly-IC exposure which was not shown by the previous group. Thus, the inhibitory effects on TSLP and CCL26 expression even after viral infection are not common among glucocorticosteroid species, but linked with the pharmacological property of BUD. In addition, the production of inflammatory chemokines that were induced by not only viral infection, but also two cytokines strongly linked to asthmatic conditions, IL-13 and IL-17A, were reduced by BUD.

Viral infections induce a variety of cytokines from bronchial epithelial cells and can trigger exacerbations of asthma [1]. TSLP, one of key cytokines, lies at the start of the inflammatory cascade in asthma, functioning as an upstream “master switch” in the epithelium [19]. Inhibition of TSLP causes marked declines in several biomarkers, including blood eosinophils, serum immunoglobulin E (IgE), and fractional exhaled nitric oxide (FeNO), and has ripple effects on multiple downstream inflammation cascades involved in asthma [20,21]. Therefore, suppressing TSLP seems like a reasonable strategy for preventing the progression of viral infection-related inflammation and currently tezepelumab, a human monoclonal antibody specific for TSLP, is studied for uncontrolled asthma [20,21].

In addition, viral infections act in concert with IL-13 to induce the release of chemokines crucial to eosinophilic inflammation, including CCL26 and CCL5, from bronchial epithelial cells [14,22]. Our results showed that incubations of bronchial epithelial cells for three hours with BUD inhibited the inductions of chemokines by poly I:C, IL-13, and IL-17A exposure, but the mechanism underlying the effect of suppression is still unclear [11]. Although other groups had raised glucocorticoids suppressed transcription factor such as nuclear factor (NF)-kappa B, we previously investigated which factors a glucocorticosteroid acts on in the TLR3 signaling pathway, but could not find clear mechanisms [11,23]. A recent report by Kim et al. showed that not only antiviral, but also anti-inflammatory activity of BUD against human rhinovirus infection is mediated via autophagy activation, suggesting several underlying mechanisms are orchestrated by BUD [24]. Mechanisms underlying these effects of BUD will be further investigated.

Like other glucocorticoids, the mechanism of action of BUD is mainly down-regulating transcription factors for various proteins via GCR, thereby inhibiting the expression of a variety of cytokines and inducible enzymes [25,26]. Every glucocorticosteroid species has its own chemical properties, especially in terms of tissue deposition/uptake and metabolic pathways. Compared to other glucocorticoids, BUD has higher water solubility, and this property seems to allow it to diffuse through mucus to reach the airway epithelium more quickly, even in mucus overproduction such as asthma or CRS [27]. This property could be one reason why its action is observed so quickly when taken under typical as-needed regimens [28].

FP was previously reported that its ability to suppress IL-6 and CXCL8 production at ~1/10th of BUD concentration, specifically when pre-incubated with A549 cells (a human pulmonary epithelial line) and human alveolar macrophages for 1.5 h before swine dust exposure [29]. Still, FP remained in the sputum for longer than BUD, whereas BUD was more rapidly absorbed, intensifying its immunosuppressive effects, and potentially making the tissue more susceptible to infection [30]. Indeed, it was reported that BUD protects against epithelial barrier dysfunction due to bacterial infection, and limits adhesion and internalization provoked by poly I:C stimulation, whereas FP exacerbates the barrier dysfunction caused by rhinovirus infection [31]. Recently, Berge et al. reported that immune defense genes in bronchial epithelial cells were altered by both BUD and FP, suggesting the similarity of both agents [32]. 

Numerous clinical studies already showed that treatment with inhaled corticosteroids reduces exacerbations in patients with asthma [33]. One such medication, BUD/FOR, has recently been shown to effectively relieve mild asthma when taken on an as-needed basis [33]. The use of inhaled corticosteroids during a viral infection is still controversial because of the inhibition of the innate immunity of airway epithelial cells. A recent study by Thomas et al. showed enhanced replication of HRV16 by the addition of glucocorticosteroids [34]. It was reported by Yamaya et al. that budesonide inhibited HRV14 infection and cytokine production in primary cultures of human tracheal epithelial cells [35]. Our current study did not find the alteration of the viral load by the addition of BUD to epithelial cells and Bochkov et al. showed no alteration of viral load with or without BUD [36]. One of the limitations of our study is that both the prior and late addition of BUD were not tested. Therefore, the effect of BUD may be overexaggerated when compared to real-world data, but our current data can be interpreted that the late addition of BUD was applied to the glucocorticosteroid-naïve asthma patient. Our current data only shows inhibition of TSLP and CCL26 from airway epithelial cells. From our past findings, other Th2-related chemokines may also be down-regulated by BUD, but clearly, further study is needed to clear our limitations. Currently, we are preparing a study to clarify these surrounding issues to understand the molecular mechanisms.

## 5. Conclusions

BUD significantly suppressed poly I:C-induced TSLP and CCL26 expression even after viral exposure, and this pharmacological property seems to be unique to BUD. In addition, the production of inflammatory chemokines normally triggered by IL-13 and IL-17A, which are strongly linked to not only viral infection but also asthmatic condition, was reduced by the late addition of BUD. These results indicate that BUD has anti-inflammatory effects in treating chronic Th2 allergy dominant airway disease, such as asthma, even in cases with suspected acute exacerbation of asthma by viral infection.
